# Asiaticoside reverses M2 phenotype macrophage polarization-evoked osteosarcoma cell malignant behaviour by TRAF6/NF-κB inhibition

**DOI:** 10.1080/13880209.2022.2109688

**Published:** 2022-08-22

**Authors:** Dang-ke Li, Guang-hui Wang

**Affiliations:** Department of Orthopaedics, Qilu Hospital of Shandong University, Qingdao, China

**Keywords:** Tumour necrosis factor receptor-associated factor 6, viability, invasion, THP-1

## Abstract

**Context:**

M2 phenotype macrophage polarization is an attractive target for therapeutic intervention. Asiaticoside (ATS) has multiple pharmacological functions.

**Objective:**

This study investigates the effect of ATS on M2 phenotype macrophage polarization in osteosarcoma.

**Materials and methods:**

The differentiation of human THP-1 monocytes into M0 phenotype macrophages was induced by 100 nM phorbol myristate acetate for 24 h, and treated with 20 ng/mL IL-4 and 20 ng/mL IL-13 for 48 h to obtain M2 phenotype macrophages. The function of ATS on the growth and invasion was investigated by cell counting kit-8, transwell, and western blot under the co-culture of M2 phenotype macrophages and osteosarcoma cells for 24 h. The mechanism of ATS on osteosarcoma was assessed using molecular experiments.

**Results:**

ATS reduced the THP-1 cell viability with an IC_50_ of 128.67 μM. Also, ATS repressed the M2 phenotype macrophage polarization induced by IL-4/IL-13, and the effect was most notably at a 40 μM dose. ATS (40 μM) restrained the growth and invasion of osteosarcoma cells induced by M2 phenotype macrophages. In addition, ATS reduced the tumour necrosis factor receptor-associated factor 6 (TRAF6)/NF-κB activity in osteosarcoma cells and the TRAF6 knockdown reduced the growth and invasion of osteosarcoma cells induced by M2 phenotype macrophages. TRAF6 (2 μg/mL) attenuated the inhibitory effect of ATS on the growth and invasion of osteosarcoma cells caused by M2 phenotype macrophages. *In vivo* studies further confirmed ATS (2.5, 5, or 10 mg/kg) repressed osteosarcoma tumour growth.

**Discussion and conclusions:**

ATS reversed M2 phenotype macrophage polarization-evoked osteosarcoma cell malignant behaviour by reducing TRAF6/NF-κB activity, suggesting ATS might be a promising drug for the clinical treatment of osteosarcoma.

## Introduction

Osteosarcoma is the most common malignant bone tumour with high metastasis and mortality (Simpson et al. 2017). Osteosarcoma usually has a poor prognosis and seriously threatens the physical and mental health of young people (Biazzo and De Paolis 2016). Unfortunately, the pathogenesis of osteosarcoma is not fully understood and effective treatment options are limited (Yang and Zhang 2013). Therefore, elucidating the novel mechanism of osteosarcoma is helpful to investigate effective treatment strategies.

Accumulated evidence clarifies that the tumour microenvironment (TME) exerts momentous roles in osteosarcoma progression (Buddingh et al. 2011). Tumour-associated macrophages (TAMs) are a pivotal component of TME and mediate the growth, angiogenesis, and stem cell-like phenotype of osteosarcoma (Garimella et al. 2013; Huang Q et al. 2021). Macrophages are a part of the innate immune system and exert a critical function in inflammatory processes, defence, repair, and metabolism, as well as a momentous factor in maintaining body stability (Ren et al. 2020). Macrophages have great functional and phenotypic plasticity, usually divided into M1 phenotype and M2 phenotype (Mosser and Edwards 2008). M2 phenotype macrophages participate in tissue remodelling, angiogenesis, wound healing, and immune regulation (Sica et al. 2006). Unlike ‘classically activated’ M1 phenotype macrophages, M2 phenotype macrophages are generally considered to have cancer-promoting functions in osteosarcoma (Shao et al. 2019). Thus, exploring strategies that repress M2 phenotype macrophage polarization has the potential to alleviate osteosarcoma.

*Centella asiatica* (L.) Urb. (Apiaceae) is an herbaceous perennial plant (Ren L et al. 2016). *C. asiatica* is a widely used traditional Chinese medicine and has anti-inflammatory and antioxidant effects (Li et al. 2021). Asiaticoside (ATS) is one of the critical components of triterpenoid extracted from *C. asiatica* and its structure is clear (He et al. 2019; Wang et al. 2020). ATS has multiple pharmacological functions, mainly containing anti-inflammatory, antioxidant, anti-fibrosis, and wound healing (Zhang et al. 2016). For example, ATS represses the proliferation and stem cell-like properties of pancreatic cancer cells by reducing the phosphorylation of p38 MAPK and NF-κB/P65 in pancreatic cancer cells (He Y et al. 2021). Crucially, a recent study verifies that ATS accelerates tissue regeneration by mediating the M2 phenotype macrophage polarization (Huang et al. 2020). However, the role and mechanism of ATS in osteosarcoma remain unclear.

In this study, we preliminarily defined the dose range of ATS with a non-toxic effect on THP-1 cells. We then confirmed that ATS repressed M2 phenotype macrophage polarization induced by IL-4/IL-13, and the dose of 40 μM had the best effect. On this basis, the potential mechanism of ATS in repressing the M2 phenotype macrophage polarization was further investigated in osteosarcoma. These findings may provide novel drugs and insights for the clinical treatment of osteosarcoma.

## Materials and methods

### Cell culture and different treatments

THP-1 and U2OS cells were from the American Type Culture Collection (ATCC, Manassas, USA). THP-1 cells were cultured in RPMI 1640 medium containing 10% foetal bovine serum (FBS, Beyotime, Shanghai, China). U2OS cells were grown in ATCC-formulated McCoy’s 5a Medium Modified (ATCC, #30-2007) with 10% FBS. All cells were cultured at 37 °C with 5% CO_2_.

THP-1 cells were incubated with 100 nM phorbol myristate acetate (PMA, Beyotime) for 24 h to induce into M0 phenotype macrophages. Cells were treated with 20 ng/mL IL-4 and 20 ng/mL IL-13 for 48 h to obtain M2 phenotype macrophages (Ma et al. 2020).

To assess the impact of ATS on the THP-1 cell viability, the cells were treated with 0, 5, 10, 20, 40, 80, and 160 μM ATS (HPLC ≥98.5%; #16830-15-2; molecular formula: C_48_H_78_O_19_; Sigma-Aldrich, Shanghai, China) for 24 h (He Y et al. 2021). To observe the influence of ATS on M2 phenotype macrophage polarization, the above-obtained M0 phenotype macrophages were induced into M2 phenotype macrophages with 20 ng/mL IL-4 and 20 ng/mL IL-13, and the cells were then treated with 5, 10, 20, and 40 μM doses of ATS for 24 h. In addition, M2 phenotype macrophages were treated with TRAF6 (0.25, 0.5, 1.0, 2.0, and 4.0 μg/mL) for 24 h. For the co-culture system, the M2 phenotype macrophages were co-cultured with U2OS cells for 24 h (Li et al. 2018).

### Cell transfection

Si-TRAF6 and si-NC (negative control) were produced by RiboBio (Guangzhou, China) and applied for knocking down TRAF6. THP-1 cells (5 × 10^5^) were added to six-well plates and cultured overnight. Then the cells were transfected with 40 nmol/L si-TRAF6 for 48 h using Lipofectamine 2000 (Thermo Fisher Scientific, MA, USA) following the standard procedure of the manufacturer. The knockdown efficiency of si-TRAF6 was evaluated using quantitative real-time PCR (qRT-PCR) and Western blot assays.

### qRT-PCR analysis

Total RNA was extracted from M2 phenotype macrophages and U2OS cells using TRIzol reagent (Solarbio, Beijing, China). RNA concentrations were determined with a UV spectrophotometer (A260/A280), the RNA was reverse transcribed into cDNA with RevertAid™ First Strand cDNA Synthesis Kits (Qiagen, NY, USA). Real-time PCR was carried out with a LightCycler^®^ 96 system (Roche, Basel, Switzerland) with SYBR Green reagents (TaKaRa, Dalian, China) following the instructions from reagent manufacturers. The relative level of different molecules was evaluated using the 2^−ΔΔCt^ method after normalization with GAPDH. The primer sequences are listed in Table 1.

**Table 1.** The sequences of all primers applied in qRT-PCR.

Gene name	Primer sequence (5′–3′)
CD86	Forward: GACCGTTGTGTGTGTTCTGG
	Reverse: GATGAGCAGCATCCAAGGA
iNOS	Forward: TCCTGGAGGAAGTGGGCCGAAG
	Reverse: CCTCCACGGGCCCGGTACTC
CCL5	Forward: TGCCCACATCAAGGAGTATTT
	Reverse: TTTCGGGTGACAAAGACGA
CD206	Forward: TCTTTGCCTTTCCCAGTCTCC
	Reverse: TGACACCCAGCGGAATTTC
CCL24	Forward: CCAGCCTTCTGTTCCTTGGTG
	Reverse: AACTGCTGGCCCTTCTTGGT
Arg-1	Forward: CAGAAGAATGGAAGAGTCAG
	Reverse: CAGATATGCAGGGAGTCAC
IL-10	Forward: GGACAACATACTGCTAACCGAC
	Reverse: TGGATCATTTCCGATAAGGCTTG
TRAF6	Forward: CATCTTCAGTTACCGACAGCTCAG
	Reverse: TGGTCGAGAATTGTAAGGCGTAT
GAPDH	Forward: ACAGCCTCAAGATCATCAGCA
	Reverse: ATGAGTCCTTCCACGATACCA

### Western blot

After the M2 phenotype macrophages, U2OS cells or osteosarcoma tissues were harvested, total protein was extracted from the cells and tissues with RIPA lysis buffer (Beyotime, proportion of RIPA for cells: 100 μL/1 × 10^6^ cells; proportion of RIPA for tissues: 200 μL/20 mg tissues) containing 1% protease inhibitor (Beyotime). The protein concentrations were then quantified using Enhanced BCA Protein Assay Kits (Beyotime) referring to the manufacturer’s protocol. Subsequently, an equal quantity of protein (25 μg) was electroblotted into PVDF membranes (Millipore, MA, USA) following electrophoretic separation on a 10% SDS-PAGE (Solarbio). After the membranes were blocked by 5% non-fat milk at room temperature (RT) for 1.5 h, the membranes were incubated with antibodies containing anti-CD206 (Solarbio, K006619P, 1:1000), anti-CCL24 (Solarbio, K006693P, 1:500), anti-Arg-1 (R&D Systems, AF5868, 1:500), anti-IL-10 (Abcam, ab133575, 1:1000), anti-Ki-67 (Abcam, ab92742, 1:5000), anti-Bcl-2 (Abcam, ab182858, 1:2000), anti-Bax (Abcam, ab182733, 1:2000), anti-VEGF (Solarbio, K009423M, 1:1000), anti-TRAF6 (Abcam, ab33915, 1:2000), anti-P65 (Abcam, ab32536, 1:1000), anti-p-P65 (Abcam, ab76302, 1:1000), anti-TGF-β (Cell Signaling Technology, #3711, 1:1000), anti-MMP-9 (Abcam, ab76003, 1:1000) and anti-GAPDH (Abcam, ab8245, 1:500) overnight at 4 °C. Next, the membranes were washed three times using 1 × TBST (Solarbio), incubated with secondary antibodies (Abcam, ab205718, 1:5000) for 2 h at RT, and then washed three times with 1 × TBST. All proteins were visualized by immobilon western chemiluminescent HRP substrate (Millipore). In brief, the reaction solution was evenly added to the membranes (protein surface), and then the membranes (protein surface) were gently put into the colorimeter. The film was scanned or photographed and the band intensity was assessed using Image Lab 3.0 software (Bio-Rad Laboratories).

### Cell Counting Kit-8 analysis

The U2OS cell viability was evaluated using the Cell Counting Kit-8 (CCK-8) assay. In detail, U2OS cells (5 × 10^3^) were seeded into 96-well plates. Following overnight culture, the cells were incubated with 10% CCK-8 reagent for 2 h at 37 °C. Finally, the absorbance was measured with a SpectraMax M3 microplate reader (Molecular Devices Corporation, Sunnyvale, CA, USA) at a wavelength of 450 nm. U2OS cell viability (%) = [A _cells with different treatments_−A_contro_]/[A _cells with no treatments_− A_control_] × 100. A _control_: no cells.

### Flow cytometry

Fluorescein isothiocyanate (FITC)-CD14, phycoerythrin (PE)-CD86, and allophycocyanin (APC)-CD206 monoclonal antibody were provided by eBioscience (CA, USA).

After the THP-1 monocytes were induced into M0 phenotype macrophages, the cells were induced into M2 phenotype macrophages and were treated with ATS. Cells were then harvested and incubated with the respective antibody solution (5 μL) for 25 min at 4 °C in the dark. Subsequently, the fluorescently labelled monocytes were assessed with a flow cytometer (Thermo Fisher Scientific). CD14 is a typical marker molecule for monocytes; M1 phenotype macrophages were positive for CD86 and M2 phenotype macrophages were positive for CD206. The results are presented as follows: CD14 + CD206+ (%).

### Transwell assay

The U2OS cell invasion was assessed using a Transwell insert (8 µm pore size, Corning Incorporated) pre-coated with Matrigel (Solarbio). U2OS cells in a serum-free culture medium were seeded into the upper chambers. The culture medium containing 10% FBS was added to the bottom chambers. Forty-eight hours later, the cells remaining in the upper chambers were removed. The cells that invaded the lower surface were fixed using 70% methanol for 25 min at RT and were stained with 0.1% crystal violet (Solarbio) for 20 min at RT. The number of invaded cells was assessed under a light microscope (Olympus, Tokyo, Japan) at a magnification of ×200.

### Enzyme-linked immunosorbent assay

Osteosarcoma cells and M2 phenotype macrophages with different treatments were harvested. Then the cell culture supernatant was obtained from the cells of each group in the media and centrifugation (1200 *g* for 5 min at 37 °C), and the contents of TGF-β, MMP-9 and IL-10 in the cell culture supernatant were determined using an Enzyme-Linked Immunosorbent Assay Kit (Abcam) following the reagent manufacturer’s standard procedure. Then the optical density (OD) values were measured by an enzyme-labelled metre (Prolong Medical Instrument, China). The standard curve is made by standard concentration and OD value. The concentrations of TGF-β, MMP-9, and IL-10 were calculated using standard curves.

### *In vivo* study

Twenty female BALB/C nude mice (5-week-old, 20–23 g) were purchased from Yison Bio (Shanghai, China). The living conditions of mice were: humidity 45–60%, 12 h light/dark cycle. All mice had free access to food and water. The animal protocol was approved by the Ethics Committee of Qilu Hospital of Shandong University (Qingdao) [(KE) 2020 (179)]. All animal assays were conducted following the ARRIVE guidelines (https://arriveguidelines.org).

Mice were randomly divided into four groups: Control (*n* = 5), ATS 2.5 mg/kg (*n* = 5), ATS 5 mg/kg (*n* = 5), and ATS 10 mg/kg (*n* = 5). U2OS cells (5 × 10^6^) were subcutaneously injected into the right hind legs of mice. After 5 days, 2.5, 5, or 10 mg/kg ATS was administered to mice by oral gavage every 2 days for 30 days (Zhou et al. 2020; He et al. 2021). The tumour volume and weight were measured every 5 days. Thirty days later, all mice were sacrificed by intra-peritoneal injection of 40 mg/kg phenobarbital (Zhou et al. 2021).

### Immunohistochemical assay

After the osteosarcoma tissues were fixed using 10% formalin (Sbjbio, Nanjing, China) and embedded in paraffin, osteosarcoma tissues were made into 5 μm sections. Then, the sections were incubated with anti-Ki67 (ab15580, 2 µg/mL, Abcam), anti-Bcl-2 (ab32124, 1/250, Abcam), anti-Bax (ab32503, 1/250, Abcam), and anti-VEGF (Solarbio, K009423M, 1/200) overnight at 4 °C. Next, sections were incubated with secondary antibody and were stained with 3,3-diaminobenzidine solution and haematoxylin. The positive cells were counted under a microscope (Olympus).

### Statistical analysis

All experimental data were assessed using SPSS18.0 software (SPSS, Chicago, IL, USA) and presented as mean ± standard deviation. Comparisons between groups were conducted using one-way analysis of variance (ANOVA) followed by Turkey’s *post hoc* test or Student’s *t*-tests. *p* < 0.05 was considered statistically significant.

## Results

### Asiaticoside represses M2 phenotype macrophage polarization induced by IL-4/IL-13

**Figure 1.**
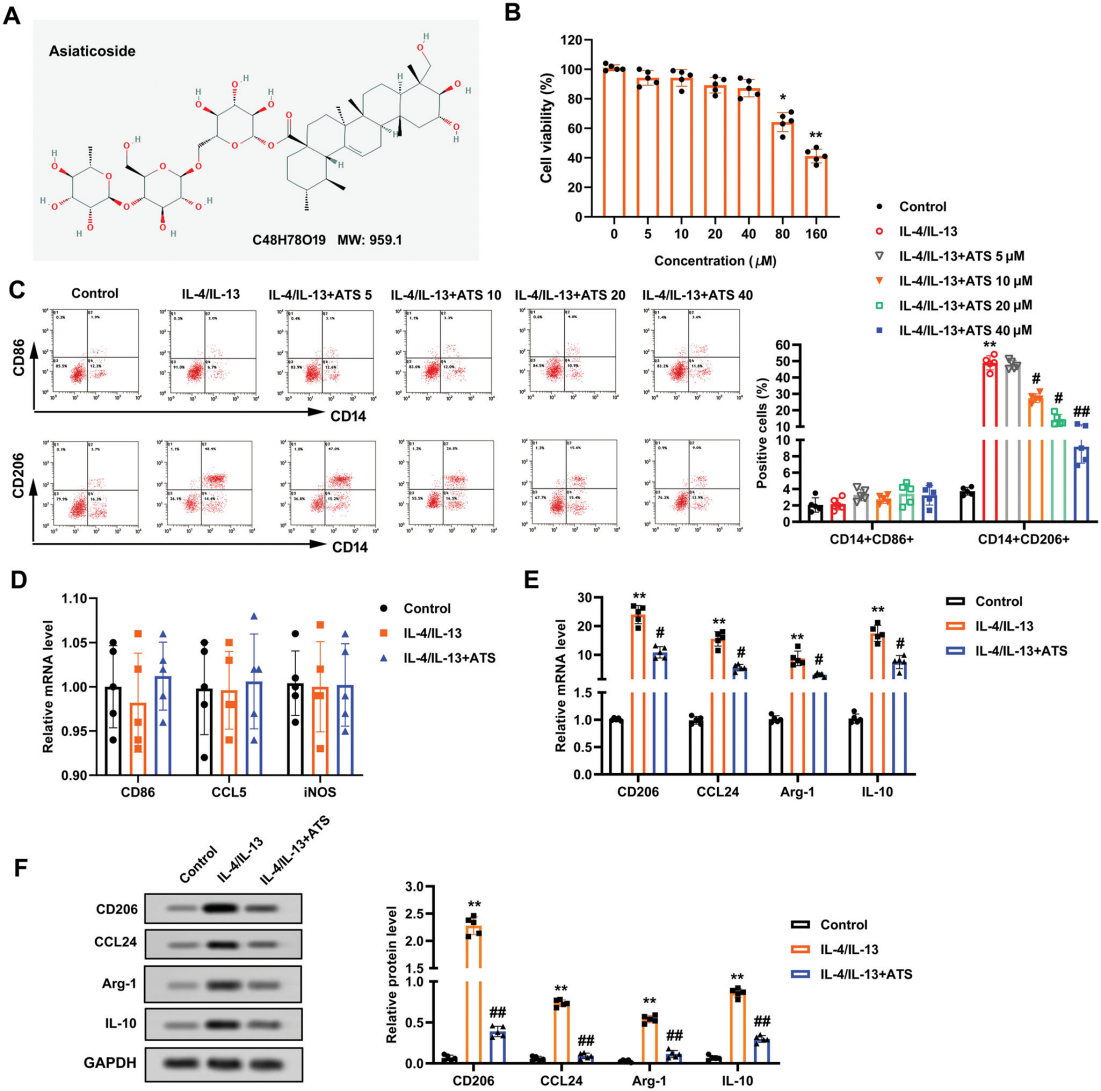
Inhibition of asiaticoside on the M2 phenotype macrophage polarization induced by IL-4/IL-13. (A) The molecular structure of asiaticoside (ATS). (B) THP-1 cells were treated with 0, 5, 10, 20, 40, 80, and 160 μM ATS for 24 h. The viability of THP-1 cells was determined using Cell Counting Kit-8 (CCK-8) analysis. (C) After the THP-1 monocytes were induced into M0 phenotype macrophages by incubation with 100 nM phorbol myristate acetate (PMA) for 24 h, the cells were induced to polarize to M2 phenotype macrophages with IL-4/IL-13, and the cells were treated with 5, 10, 20, and 40 μM doses of ATS for 24 h. The positive rates of CD14 + CD86+ cells (M1 phenotype) and CD14 + CD206+ cells (M2 phenotype) were assessed by flow cytometry. (D) After the THP-1 monocytes were induced into M0 phenotype macrophages by incubation with 100 nM PMA for 24 h, the cells were induced to polarize to M2 phenotype macrophages with IL-4/IL-13, and the cells were treated with 40 μM ATS for 24 h. The mRNA levels of M1 phenotype macrophages markers CD86, iNOS, and CCL5 were measured using quantitative real-time PCR (qRT-PCR). (E) Analysis of the M2 phenotype macrophages markers CD206, CCL24, Arg-1, and IL-10 expression by qRT-PCR. (F) The protein levels of CD206, CCL24, Arg-1, and IL-10 were quantified using Western blot. *p < 0.05, **p < 0.01 vs. control. ^#^*p* < 0.05, ^##^*p* < 0.01 vs. IL-4/IL-13. Each assay was conducted in triplicate.

To examine the impact of ATS on the M2 phenotype macrophage polarization, the toxicity of ATS on THP-1 cells was evaluated. The molecular structure of ATS is displayed in Figure 1(A). CCK-8 assay clarified that the viability of THP-1 cells was prominently reduced by ∼40% when the ATS concentration was ≥80 μM. Also, ATS restrained the THP-1 cell viability with an IC_50_ of 128.67 μM (Figure 1(B)). Therefore, the doses of 5, 10, 20, and 40 μM ATS were selected for follow-up studies.

During the induction of M2 phenotype macrophage polarization, the cells were treated with the above doses of ATS. Flow cytometry results revealed that ATS had no notable impact on the positive rate of CD14 + CD86+ cells (M1 phenotype), and decreased the positive rate of CD14 + CD206+ cells (M2 phenotype) induced by IL-4/IL-13 in a dose-dependent manner, and the effect was most notably at 40 μM dose (Figure 1(C)). Thus, 40 μM ATS was chosen for subsequent assays. CD86, iNOS, and CCL5 are markers for M1 phenotype macrophages, and CD206, CCL24, Arg-1, IL-10 are markers for M2 phenotype macrophages (Plastira et al. 2016; Wang X et al. 2021). As exhibited in Figure 1(D,E), the expression of CD86, iNOS, and CCL5 had no remarkable changes after the ATS treatment, while the expression of CD206, CCL24, Arg-1, IL-10 was decreased. Meanwhile, Western blot analysis demonstrated that the ATS treatment reduced the protein levels of CD206, CCL24, Arg-1, and IL-10 (Figure 1(F)). In summary, ATS restrained the M2 phenotype macrophage polarization induced by IL-4/IL-13.

### Asiaticoside reduces the growth and invasion of osteosarcoma cells induced by M2 phenotype macrophages

**Figure 2.**
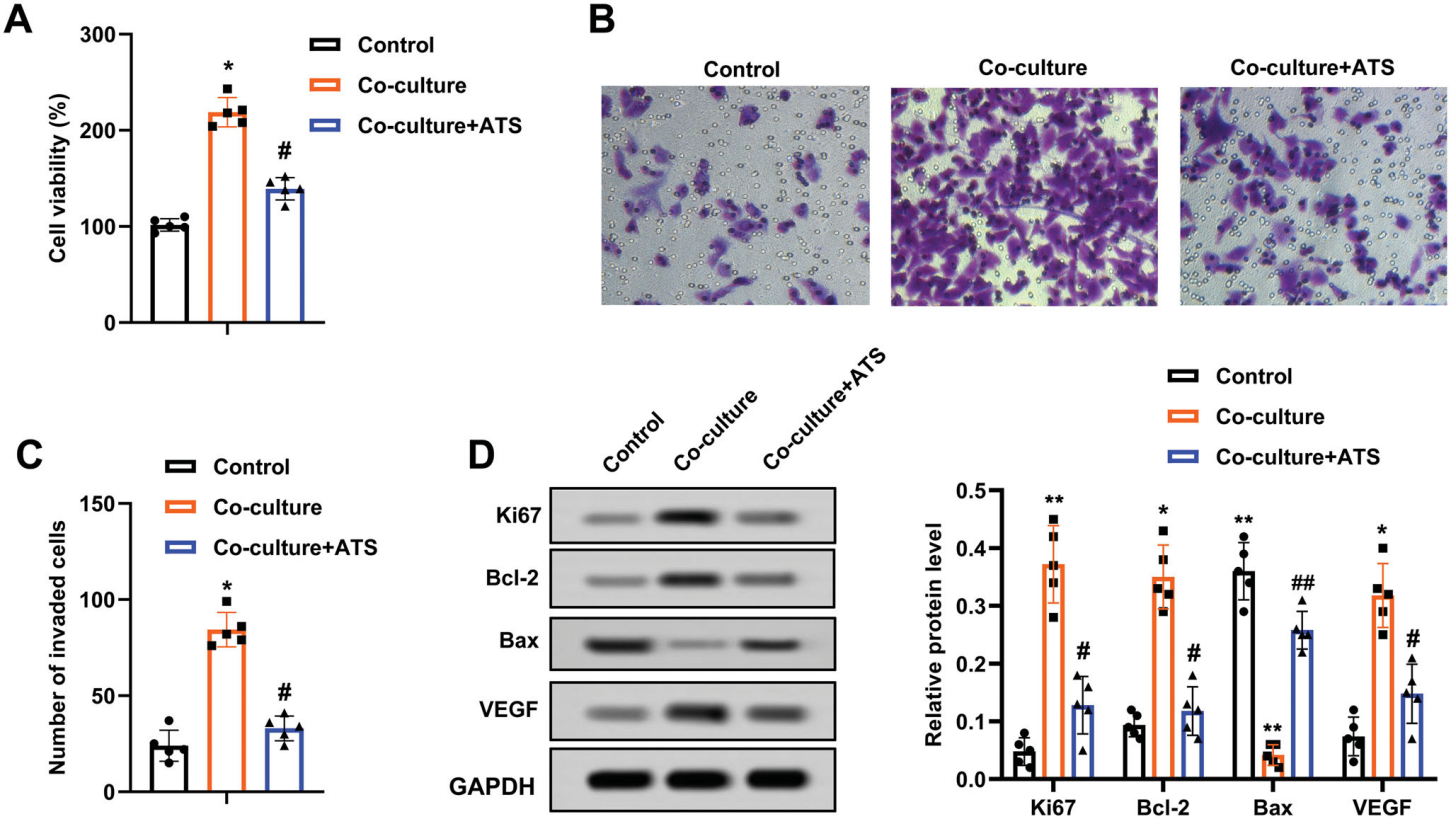
Impact of asiaticoside on the growth and invasion of osteosarcoma cells induced by M2 phenotype macrophages. M2 phenotype macrophages induced by IL-4/IL-13 were co-cultured with osteosarcoma cells, and the cells were treated with 40 μM ATS for 24 h. (A) The viability of U2OS cells was analyzed using a CCK-8 assay. (B) The invasion of U2OS cells was assessed by Transwell assay (magnification:200). (C) The quantitative results of cell invasion. (D) The protein levels of Ki-67, Bcl-2, Bax, and vascular endothelial growth factor (VEGF) were measured using Western blot. *p < 0.05, **p < 0.01 vs. control. ^#^*p* < 0.05, ^##^*p* < 0.01 vs. co-culture. Each assay was conducted in triplicate.

Considering that ATS restrained M2 phenotype macrophage polarization and M2 phenotype macrophages accelerate osteosarcoma progression (Wolf-Dennen et al. 2020), we attempted to clarify whether ATS influenced the growth and invasion of osteosarcoma cells through repressing the M2 phenotype macrophage polarization. M2 phenotype macrophages were co-cultured with U2OS cells, and the cells were treated with ATS. As displayed in Figure 2(A), the co-culture of M2 phenotype macrophages enhanced the viability of U2OS cells, while this trend was partially reversed by the ATS treatment. Meanwhile, the Transwell assay demonstrated that the U2OS cell invasion was increased in the co-culture group in comparison with the control group, while the ATS treatment partially reversed this increase (Figure 2(B)). The quantitative results of cell invasion are presented in Figure 2(C).

Ki-67 is a proliferation-related protein, and Bcl-2, Bax are apoptosis-related proteins (Zhu et al. 2020); vascular endothelial growth factor (VEGF) accelerates angiogenesis (Karami et al. 2020). Western blot analysis clarified that the protein levels of Ki-67, Bcl-2, VEGF were elevated, and Bax was decreased in U2OS cells after the co-culture of M2 phenotype macrophages, and these responses were partially reversed by the ATS treatment (Figure 2(D)). Conclusively, ATS repressed the growth and invasion of U2OS cells induced by M2 phenotype macrophages.

### Asiaticoside reduces the TRAF6/NF-κB activity in osteosarcoma cells

**Figure 3.**
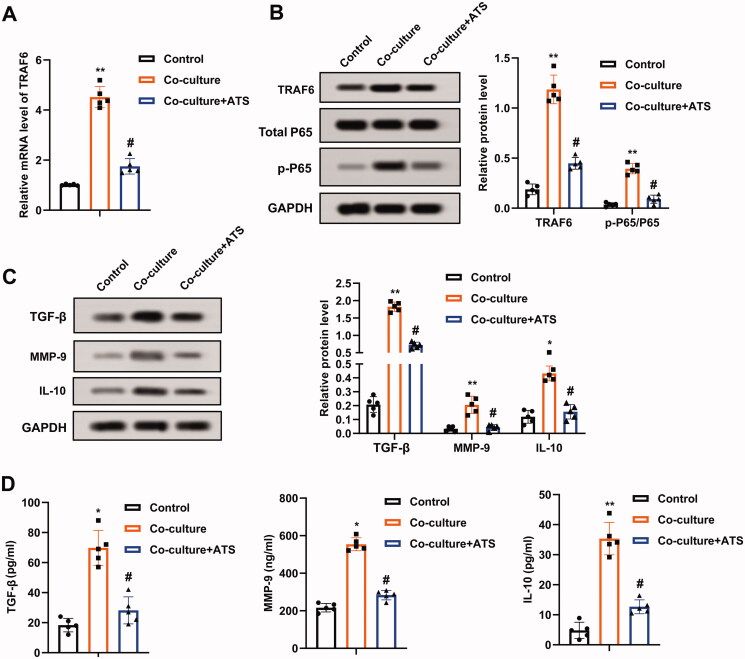
Influence of asiaticoside on the TRAF6/NF-κB activity in osteosarcoma cells. M2 phenotype macrophages induced by IL-4/IL-13 were co-cultured with osteosarcoma cells, and the cells were treated with 40 μM ATS for 24 h. (A) The TRAF6 mRNA level in U2OS cells was determined using qRT-PCR. (B) The protein levels of TRAF6, Total P65, and p-P65 were measured by Western blot, and the quantification of TRAF6 and p-P65/P65. (C) Analysis of TGF-β, MMP-9, and IL-10 protein levels using Western blot, and the quantification of TGF-β, MMP-9, and IL-10 protein levels was displayed. (D) The contents of TGF-β, MMP-9, and IL-10 in the supernatant of U2OS cell culture were detected by enzyme-linked immunosorbent assay (ELISA). *p < 0.05, **p < 0.01 vs. control. ^#^*p* < 0.05 vs. co-culture. Each assay was conducted in triplicate.

A previous study corroborates that TRAF6 mediates osteosarcoma development (Meng et al. 2018), and the TRAF6/NF-κB axis exerts pivotal functions in multiple diseases (Wang et al. 2019; Wen et al. 2022). Thus, this study sought to clarify whether ATS regulated osteosarcoma progression through the TRAF6/NF-κB axis. Firstly, the TRAF6 mRNA level was evaluated, and the data confirmed that the co-culture of M2 phenotype macrophages increased the TRAF6 expression by more than four times, and this trend was partially reversed after the ATS treatment (Figure 3(A)). Meanwhile, the TRAF6 and p-P65 protein levels were elevated after the co-culture of M2 phenotype macrophages, while the ATS treatment partially reversed this impact (Figure 3(B)).

TGF-β, MMP-9, and IL-10 are pivotal molecules interrelated to the NF-κB pathway (Araújo et al. 2013; Wu et al. 2018). As displayed in Figure 3(C), the co-culture of M2 phenotype macrophages elevated the TGF-β, MMP-9, and IL-10 protein levels, and this trend was partially reversed by the ATS treatment. Moreover, the contents of TGF-β, MMP-9, and IL-10 in the U2OS cell culture supernatant exhibited the same trend (Figure 3(D)). In general, ATS decreased the TRAF6/NF-κB activity in osteosarcoma cells.

### Interference with TRAF6 inhibits the growth and invasion of osteosarcoma cells induced by M2 phenotype macrophages

**Figure 4.**
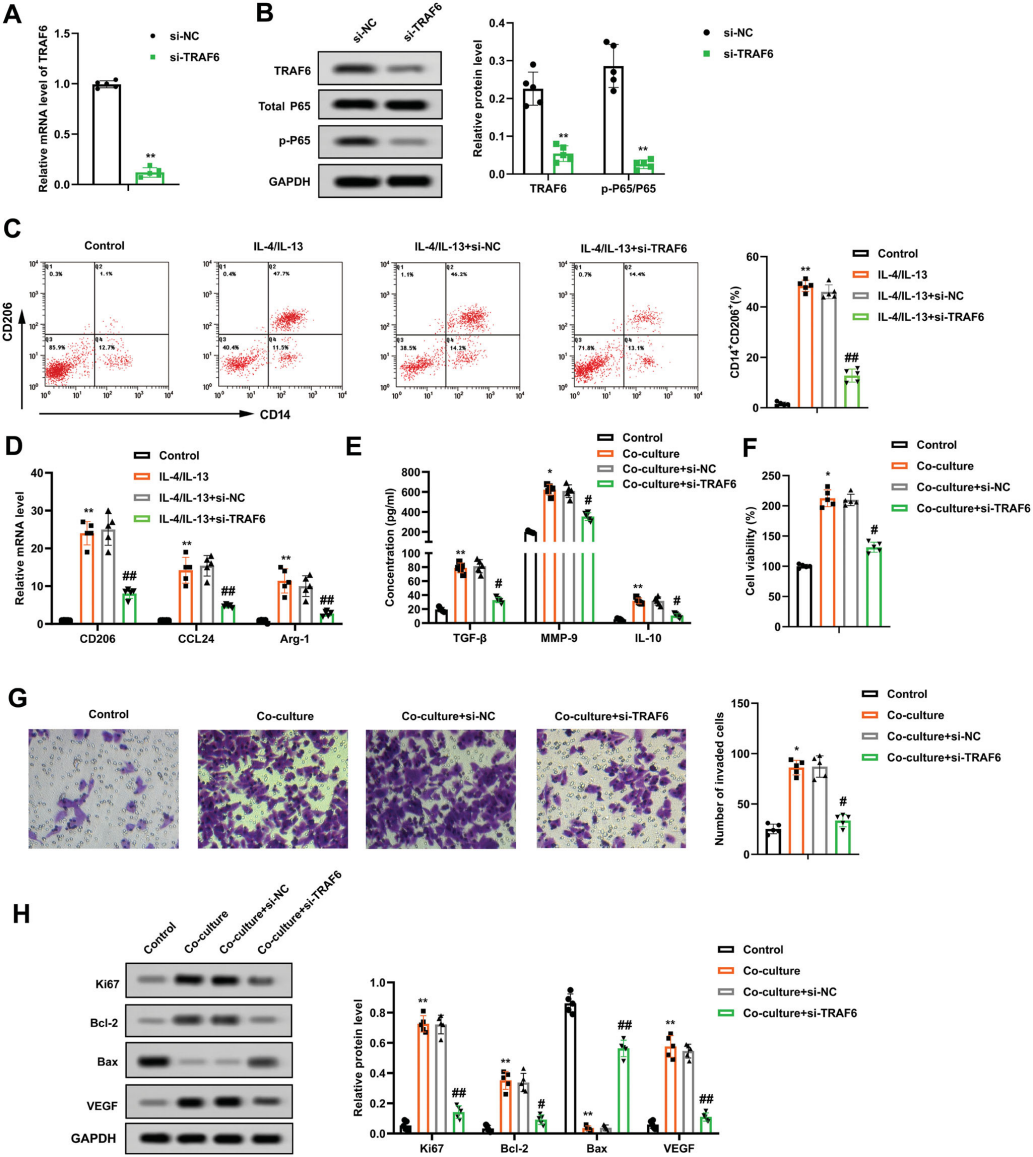
Regulation of TRAF6 on the growth and invasion of osteosarcoma cells induced by M2 phenotype macrophages. Si-TRAF6 or si-NC was transfected into THP-1 cells for 48 h. (A and B) The transfection efficiency of si-TRAF6 was confirmed using qRT-PCR and Western blot, and the protein quantification of TRAF6 and p-P65/P65 was presented. (C) The positive rate of M2 phenotype macrophages was determined by flow cytometry. (D) The mRNA levels of CD206, CCL24, and Arg-1 were assessed using qRT-PCR. (E) Comparison of TGF-β, MMP-9, and IL-10 contents by ELISA. (F) M2 phenotype macrophages transfected with si-TRAF6 were co-cultured with osteosarcoma cells. The viability of U2OS cells was measured using a CCK-8 assay. (G) Analysis of the U2OS cell invasion by Transwell and the quantitative results of cell invasion. (H) Detection of the Ki-67, Bcl-2, Bax, and VEGF protein levels using Western blot. **p < 0.01 vs. si-NC. *p < 0.05, **p < 0.01 vs. control. ^#^*p* < 0.05, ^##^*p* < 0.01 vs. co-culture + si-NC. Each assay was conducted in triplicate. NC, negative control.

Subsequently, the impact of TRAF6 on the growth and invasion of osteosarcoma cells induced by M2 phenotype macrophage was evaluated. Si-TRAF6 was transfected into THP-1 cells, and the transfection efficiency was verified by qRT-PCR and Western blot (Figure 4(A,B)). Furthermore, the p-P65 protein level decreased after the TRAF6 knockdown, and the protein quantification of TRAF6 and p-P65/P65 is displayed in Figure 4(B). On this basis, THP-1 cells transfected with si-TRAF6 were treated with PMA, and then induced into M2 phenotype macrophages with IL-4/IL-13. The positive rate of M2 phenotype macrophages was assessed and the data authenticated that the IL-4/IL-13 treatment increased the positive rate of CD14 + CD206+ cells, while this impact was partially reversed after the transfection of si-TRAF6 (Figure 4(C)), implying that the TRAF6 knockdown reduced the positive rate of M2 phenotype macrophages and the positive rates fell by nearly three-quarters. Meanwhile, the transfection of si-TRAF6 decreased the expression of CD206, CCL24, and Arg-1 (Figure 4(D)). Meanwhile, ELISA results further corroborated that the contents of TGF-β, MMP-9, and IL-10 were reduced after the TRAF6 knockdown (Figure 4(E)).

Moreover, the M2 phenotype macrophages transfected with si-TRAF6 were co-cultured with osteosarcoma cells. As exhibited in Figure 4(F), knocking down TRAF6 weakened the viability of U2OS cells. The invasion of U2OS cells had similar trends (Figure 4(G)). The protein levels of Ki-67, Bcl-2, Bax, and VEGF were determined and the data indicated that Ki-67, Bcl-2, VEGF were decreased, and Bax was increased after the TRAF6 knockdown (Figure 4(H)). All in all, the TRAF6 knockdown restrained the growth and invasion of osteosarcoma cells induced by M2 phenotype macrophages.

### TRAF6 attenuates the inhibitory effect of asiaticoside on the growth and invasion of osteosarcoma cells induced by M2 phenotype macrophages

**Figure 5.**
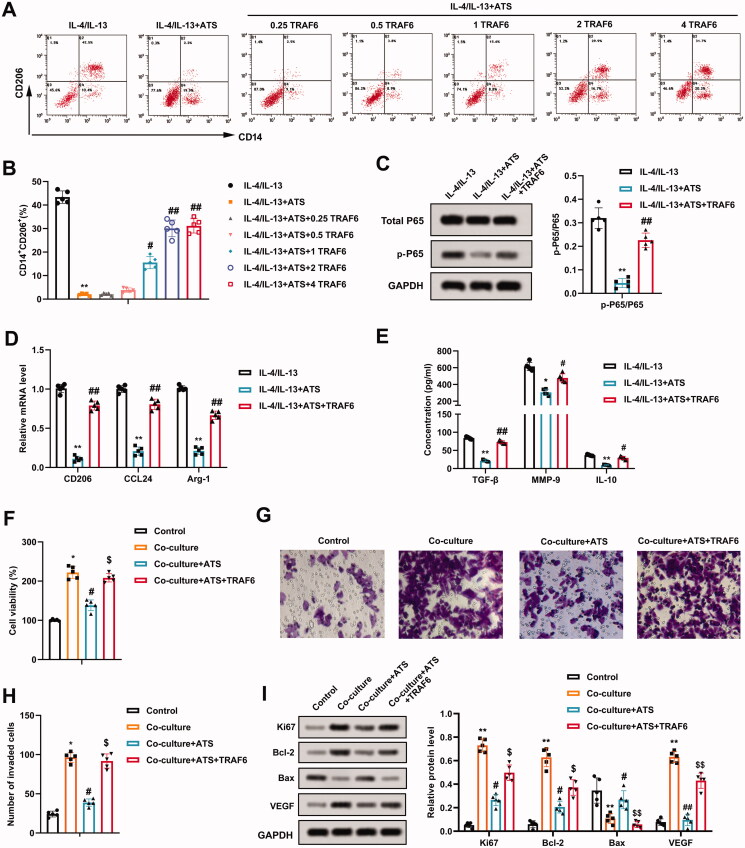
Impact of TRAF6 on the inhibitory effect of asiaticoside on the growth and invasion of osteosarcoma cells induced by M2 phenotype macrophages. IL-4/IL-13-induced M2 phenotype macrophages were treated with 40 μM ATS and TRAF6 (0.25, 0.5, 1.0, 2.0, and 4.0 μg/mL). (A and B) The positive rate of M2 phenotype macrophages was assessed using flow cytometry. (C) The total P65 and p-P65 protein levels were determined by Western blot. (D) Detection of the CD206, CCL24, and Arg-1 expressions using qRT-PCR. (E) The TGF-β, MMP-9, and IL-10 contents were measured by ELISA. (F) M2 phenotype macrophages induced by IL-4/IL-13 were co-cultured with osteosarcoma cells, and the cells were treated with 40 μM ATS and 2 μg/mL TRAF6. Comparison of the U2OS cell viability using CCK-8 assay. (G and H) The invasion of U2OS cells was assessed by Transwell and the quantitative results of cell invasion (magnification: 200). (I) Comparison of the Ki-67, Bcl-2, Bax, and VEGF protein levels using Western blot. **p < 0.01 vs. IL-4/IL-13. *p < 0.05, **p < 0.01 vs. control. ^#^*p* < 0.05, ^##^*p* < 0.01 vs. IL-4/IL-13 + ATS or co-culture. ^$^*p* < 0.05, ^$$^*p* < 0.01 vs. co-culture + ATS. Each assay was conducted in triplicate. NC, negative control.

IL-4/IL-13-induced M2 phenotype macrophages were treated with ATS and different concentrations of TRAF6. From the results of flow cytometry, 2 and 4 μg/mL TRAF6 dramatically elevated the positive rate of M2 phenotype macrophages and there was no notable difference between 2 and 4 μg/mL doses (Figure 5(A,B)). Thus, 2 μg/mL was selected as the TRAF6 dose in subsequent assays. Meanwhile, the p-P65 protein level was elevated after the TRAF6 treatment (Figure 5(C)). The mRNA levels of CD206, CCL24 and Arg-1 were determined and the results expounded that the TRAF6 treatment elevated the CD206, CCL24 and Arg-1 expressions (Figure 5(D)). The TGF-β, MMP-9, and IL-10 contents displayed the same trend (Figure 5(E)).

M2 phenotype macrophages were co-cultured with osteosarcoma cells, and the cells were treated with ATS and TRAF6. CCK-8 analysis verified that the viability of U2OS cells was weakened after the ATS treatment, while this response was partially reversed by the TRAF6 treatment (Figure 5(F)). Meanwhile, the invasion of U2OS cells exhibited a similar trend (Figure 5(G,H)). As shown in Figure 5(I), the ATS treatment down-regulated the protein levels of Ki-67, Bcl-2, VEGF, and up-regulated Bax, while these effects were partially reversed after the TRAF6 treatment. Overall, TRAF6 attenuated the inhibitory effect of ATS on the growth and invasion of osteosarcoma cells induced by M2 phenotype macrophages.

### *In vivo* validation of the palliative effect of asiaticoside on osteosarcoma

**Figure 6.**
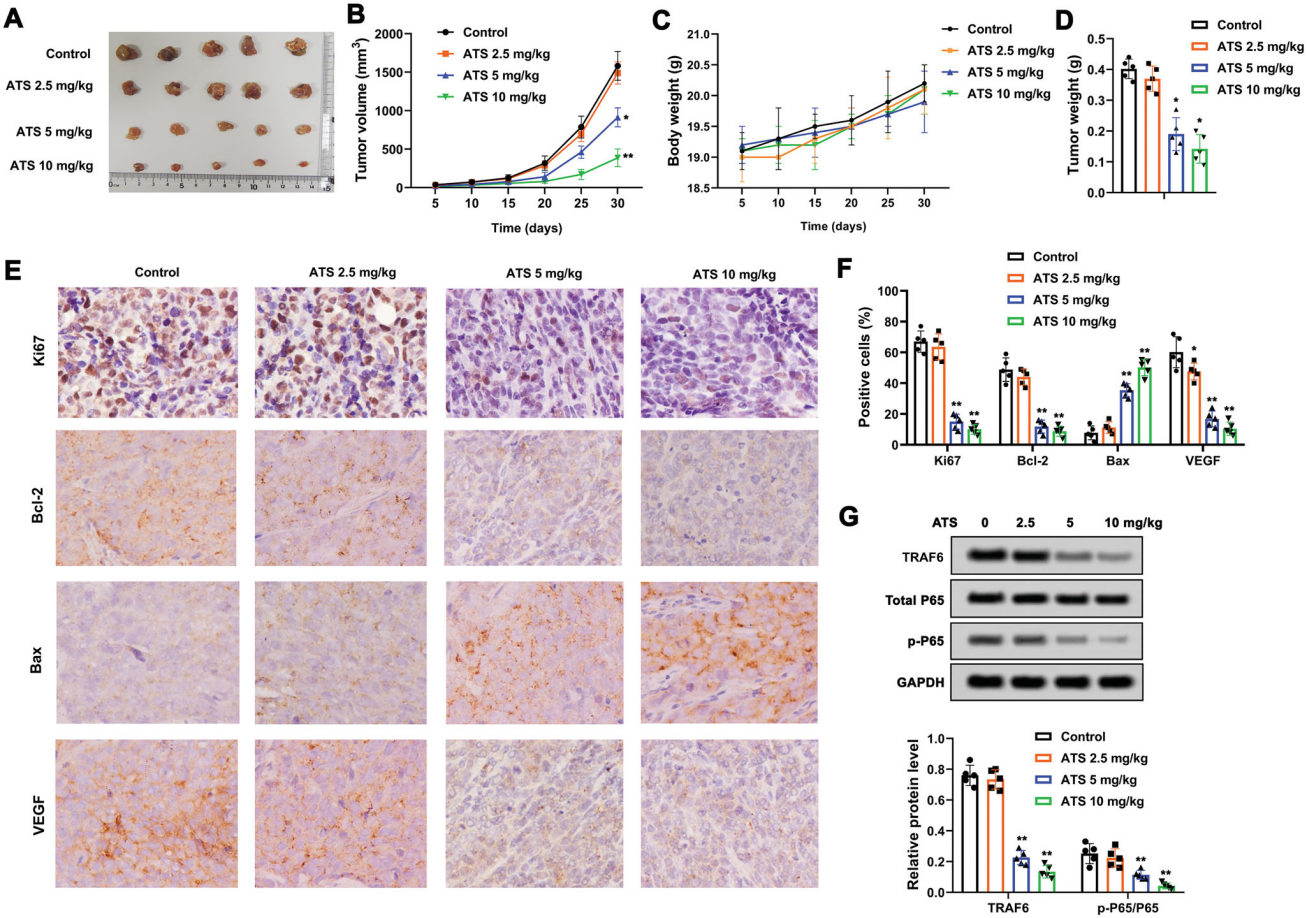
Influence of Asiaticoside on osteosarcoma *in vivo*. U2OS cells (5 × 10^6^) were subcutaneously injected into the right hind legs of mice. After 5 days, 2.5, 5, or 10 mg/kg ATS was administered to mice by oral gavage every 2 days for 30 days. (A and B) Analysis of the tumour growth. (C) Body weight of mice. (D) Analysis of the tumour weight of mice. (E and F) The expression of Ki-67, Bcl-2, VEGF, and Bax was measured using an immunohistochemical assay. (G) Western blot analysis of TRAF6, total P65, and p-P65 protein levels. *p < 0.05, **p < 0.01 vs. control.

We further explored the effect of ATS on osteosarcoma *in vivo*. As displayed in Figure 6(A,B), ATS reduced tumour growth, and tumour growth became slower as the dose increased. Besides, the different doses of ATS had no obvious influence on the body weight of mice (Figure 6(C)), while ATS decreased the tumour weight of mice (Figure 6(D)). Moreover, immunohistochemical results revealed that ATS decreased the expressions of Ki-67, Bcl-2, VEGF, and increased the Bax expression (Figure 6(E,F)). Furthermore, Western blot analysis indicated that ATS decreased the protein levels of TRAF6 and p-P65/P65 (Figure 6(G)). To sum up, ATS repressed osteosarcoma growth *in vivo*.

## Discussion

Although osteosarcoma treatment has made some progress in recent years, its prognosis is still poor (Zheng et al. 2021). Thus, searching for effective treatment is crucial. Given the cancer-promoting function of M2 phenotype macrophages (Weng et al. 2019) and ATS mediates the M2 phenotype macrophage polarization (Huang et al. 2020), this study aimed to reveal the function and mechanism of ATS in the M2 phenotype macrophage polarization in osteosarcoma. Functionally, ATS repressed the M2 phenotype macrophage polarization, and restrained the growth and invasion of U2OS cells induced by M2 phenotype macrophages. Mechanistically, ATS reduced the TRAF6/NF-κB activity in osteosarcoma cells and the TRAF6 knockdown reduced the growth and invasion of osteosarcoma cells induced by M2 phenotype macrophages. Furthermore, TRAF6 attenuated the inhibitory effect of ATS on the growth and invasion of osteosarcoma cells induced by M2 phenotype macrophages. Meanwhile, ATS had an alleviated effect on osteosarcoma *in vivo*. The completion of this study might provide new drugs and insights for osteosarcoma treatment.

ATS is the main monomer component of *C. asiatica* and has momentous regulatory functions in various human diseases, mainly including inflammatory lung disease and nervous system disease (Luo et al. 2015; Qiu et al. 2015). ATS restrains pancreatic cancer cell proliferation and facilitates cell apoptosis, thereby alleviating pancreatic cancer (He Y et al. 2021). However, to our knowledge, ATS function on osteosarcoma is still unknown. Previous studies have reported that activated macrophages are usually divided into two types: M1 phenotype macrophages and M2 phenotype macrophages, and M2 phenotype macrophages mainly mediate anti-inflammatory response (Yunna et al. 2020). Thus, regulating the activation state of macrophages to ameliorate the inflammatory environment is one of the effective strategies to relieve human diseases. Previous studies confirm that M2 phenotype macrophages exert momentous functions in osteosarcoma and ATS mediates the M2 phenotype macrophage polarization (Shao et al. 2019; Huang et al. 2020). Similar to these findings, our research demonstrated that ATS repressed the M2 phenotype macrophage polarization, and restrained the growth and invasion of U2OS cells induced by M2 phenotype macrophages.

Tumour necrosis factor receptor-associated factor 6 (TRAF6) belongs to the tumour necrosis factor receptor-associated factor (TRAF) protein family and is critical for regulating toll-like receptor (TLR)-mediated signalling (Akira et al. 2001). TRAF6 is located at the centre of signal aggregation induced by toll-like receptor and tumour necrosis factor receptor families and is widely interrelated to inflammatory and immune responses mainly through mediating inflammatory and apoptotic signalling pathways (Inoue et al. 2007). TRAF6 binds to NF-κB-induced kinase and other signalling molecules and is the critical activator of the NF-κB signalling pathway (Ye et al. 2002). TRAF6 exerts pivotal functions in tumorigenesis mainly by the regulation of the NF-κB signalling pathway. For instance, colorectal cancer patients with high expression of TRAF6 have poor survival, and TRAF6 further aggravates colorectal cancer by activating the TRAF6/NF-κB signalling pathway (Zhu et al. 2019); GTPase M accelerates the M2 phenotype macrophage polarization in gliomas through the TRAF6/NF-κB pathway, thereby accelerating the gliomas progression (Xu et al. 2019). Similar to the above conclusions, this research confirmed that ATS decreased the protein levels of TRAF6, and reduced the TRAF6/NF-κB activity in osteosarcoma cells, prompting that ATS might have a protective function in osteosarcoma through the inactivation of the TRAF6/NF-κB signalling pathway.

Accumulated evidence suggests that TRAF6 functions as an oncogene in various malignancies including osteosarcoma. For example, the expression of TRAF6 in tumour tissues of patients with pulmonary metastatic osteosarcoma is higher than that of patients without pulmonary metastasis, and the interference with TRAF6 represses the osteosarcoma cell proliferation (Meng et al. 2012); the targeted reduction of TRAF6 expression hinders the osteosarcoma progression, prompting that TRAF6 might be a promising novel biomarker for the diagnosis and treatment of osteosarcoma (Guo et al. 2020). Crucially, this study revealed that ATS decreased the protein level of TRAF6, implying that ATS might be a potential drug for osteosarcoma treatment. A recent study confirms that GTPase M elevates the TRAF6 expression and facilitates the transport of NF-κB to the nucleus, thus accelerating the M2 phenotype macrophage polarization in gliomas (Xu et al. 2019). On this basis, the current research demonstrated that the TRAF6 knockdown repressed the growth and invasion of osteosarcoma cells induced by M2 phenotype macrophages. Meanwhile, this research further authenticated that TRAF6 attenuated the inhibitory effect of ATS on the growth and invasion of osteosarcoma cells induced by M2 phenotype macrophages.

## Conclusions

This study provides evidence that ATS reduced M2 phenotype macrophage polarization induced by IL-4/IL-13, and repressed the growth and invasion of U2OS cells induced by M2 phenotype macrophages. In addition, the mechanistic results further suggest that ATS decreased the activity of TRAF6/NF-κB in osteosarcoma cells, and TRAF6 weakened the inhibitory effect of ATS on the growth and invasion of osteosarcoma cells caused by M2 phenotype macrophages. Furthermore, ATS relieved osteosarcoma *in vivo*. The data of this study suggest that ATS might be a promising drug for the treatment of osteosarcoma. The limitation of this study was the effect of ATS on macrophage polarization after co-injection of osteosarcoma cells and macrophages into mice was not involved. The study of TME after the co-transfection of macrophages and tumour cells in nude mice is a major undertaking. At present, we are in the early stages of this research, and the studies that would be carried out mainly include the construction of the animal model of co-injection of osteosarcoma cells and macrophages. After the model establishment, in addition to the conventional detection of tumour growth, apoptosis and invasion, serum, and monocytes should be separated and F4/80, M1-positive, and M2-positive cell markers should be screened by flow cytometry. In general, we would continue to verify the alleviating effect of ATS on osteosarcoma in animal experiments in the future to further enrich the content of this study.
